# Supplementary figures and images for: A Novel PDK1/MEK Dual Inhibitor Induces Cytoprotective Autophagy via the PDK1/Akt Signaling Pathway in Non-Small Cell Lung Cancer
Source: Pharmaceuticals (Basel). 2023 Feb 6;16(2):244. doi: 10.3390/ph16020244 (PMC9961937; doi:10.3390/ph16020244)

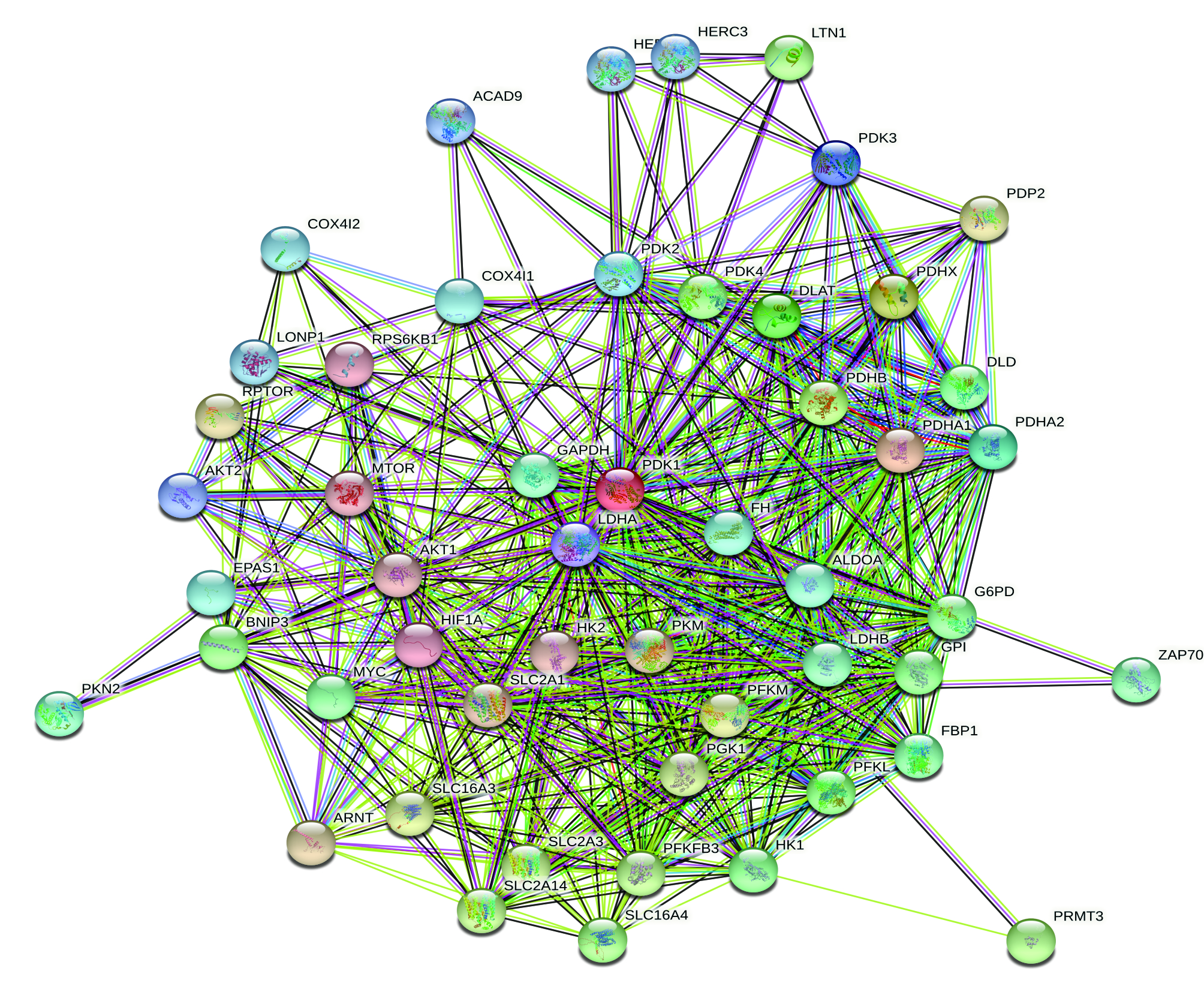

Supplement: Supplementary file 1 [file pharmaceuticals-16-00244-s001.zip › Supplementary Materials/Supplementary Figure S1.jpg]

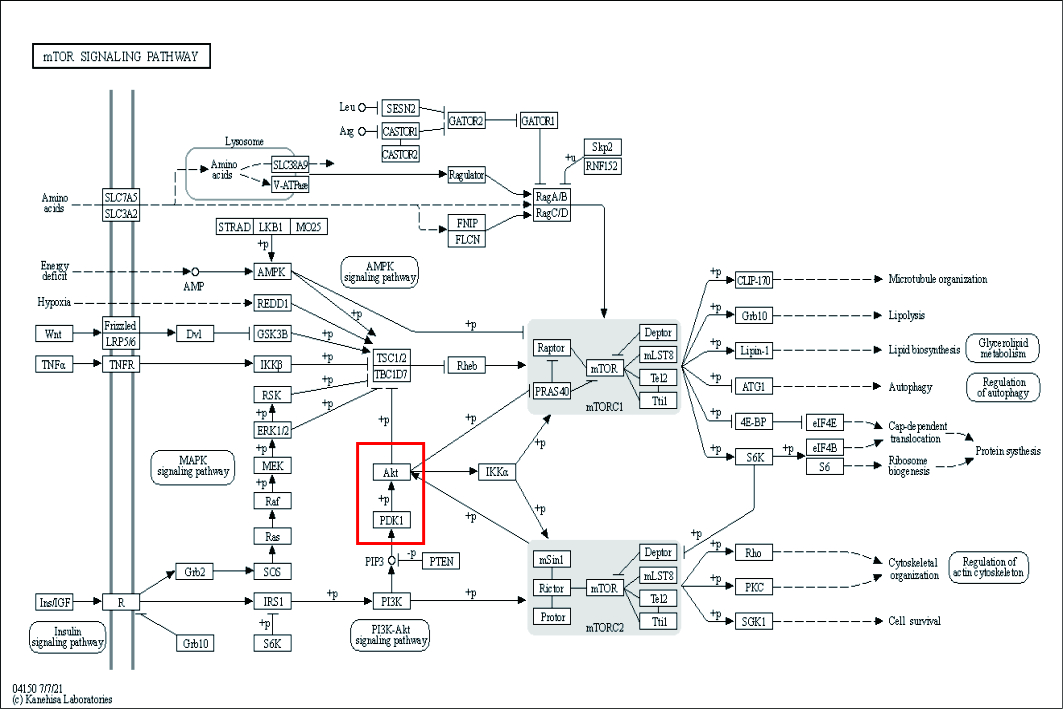

Supplement: Supplementary file 1 [file pharmaceuticals-16-00244-s001.zip › Supplementary Materials/Supplementary Figure S2.jpg]
